# Pharmacological MRI: Utility in Understanding Drug Mechanisms in Psychiatric Disorders

**DOI:** 10.1002/jmri.70160

**Published:** 2025-10-29

**Authors:** Christin Y. Sander, Tudor M. Ionescu, Mitul A. Mehta, Ottavia Dipasquale, Anouk Schrantee

**Affiliations:** ^1^ Athinoula A. Martinos Center for Biomedical Imaging, Department of Radiology Massachusetts General Hospital Charlestown Massachusetts USA; ^2^ Harvard Medical School Boston Massachusetts USA; ^3^ Department of Neuroscience and Mental Health Diseases Research Boehringer Ingelheim Pharma GmbH & Co KG Biberach an der Riss Germany; ^4^ Department of Neuroimaging Institute of Psychiatry, Psychology & Neuroscience, King's College London London UK; ^5^ Department of R&D Advanced Applications Olea Medical La Ciotat France; ^6^ Department of Radiology and Nuclear Medicine Amsterdam University Medical Center, University of Amsterdam Amsterdam the Netherlands

**Keywords:** animal models, functional imaging, pharmacological MRI, precision psychiatry, psychotropic drugs

## Abstract

Pharmacological MRI (pharmaMRI) has emerged as a powerful tool to study how psychoactive drugs affect brain function and to uncover mechanisms of drug action in psychiatric disorders. This review provides an overview of key techniques used in pharmaMRI, including BOLD‐fMRI, arterial spin labeling (ASL), and cerebral blood volume (CBV) mapping, and how they have advanced our understanding of drug‐induced changes in neural activity and connectivity. We present key advances in both preclinical and clinical pharmaMRI, highlighting its value in elucidating drug mechanisms and informing psychiatric treatment development. In preclinical studies, pharmaMRI benefits from optimized hardware, the use of contrast agents such as iron oxide nanoparticles, and precise experimental control, including invasive neuromodulation techniques (e.g., opto‐ and chemogenetics). These studies offer critical mechanistic insights into receptor‐specific and circuit‐level drug effects, supporting hypothesis‐driven translation to humans. Clinically, pharmaMRI has advanced our understanding of how compounds, such as antidepressants, antipsychotics, and stimulants, affect large‐scale brain networks and offers potential for modeling psychiatric phenotypes and predicting treatment response. Furthermore, pharmaMRI can serve as a biomarker for target engagement and refine dose selection in early‐phase trials. Despite growing interest, clinical adoption remains limited. Systematic use of both preclinical and clinical pharmaMRI could accelerate drug development, support precision psychiatry, and deepen understanding of treatment mechanisms. Finally, open science practices and large‐scale datasets can help contextualize drug effects within broader neural and molecular frameworks. While these developments present some challenges, they position pharmaMRI as a critical bridge between molecular mechanisms and systems‐level brain function in psychiatry.

**Evidence Level:**

N/A.

**Technical Efficacy:**

Stage 1.

## 1 Introduction

Pharmacological magnetic resonance imaging (pharmaMRI) has emerged as a powerful tool for investigating how drugs with relevance for psychiatry modulate brain function, offering insights into both therapeutic mechanisms and the neurochemical underpinnings of mental health disorders. By noninvasively measuring hemodynamic responses to pharmacologic challenges, pharmaMRI links drug‐induced changes in neural activity to functional brain networks, complementing traditional molecular imaging approaches like positron emission tomography (PET). This capability is particularly valuable in psychiatry, where drugs often exert complex effects across distributed circuits implicated in multiple domains of motivation, reward, and cognition.

The primary goal of this review is to provide an overview of the current state‐of‐the‐art in pharmaMRI and its contributions to the study of drug mechanisms in psychiatry. By measuring dynamic, dose‐dependent, and time‐resolved brain responses to pharmacological agents, pharmaMRI enables precise mapping of central nervous system targets and functional engagement. This ability supports translational research across species and phases of drug development, from mechanistic studies in animal models to proof‐of‐concept trials in humans. As such, pharmaMRI has expanded our understanding of how different classes of drugs, including antipsychotics, antidepressants, and stimulants, affect brain function [1, 2]. For the purposes of this review, we refer to pharmaMRI as the study of acute or sub‐acute physiological effects of a drug—those occurring immediately after administration or within a short time frame (typically the same day or the following day)—rather than changes resulting from prolonged treatment. We reserve the abbreviation phMRI to specifically refer to studies that measure the acute effects of a drug challenge during dynamic MR acquisitions.

To contextualize these contributions, we first outline the core principles of functional imaging techniques employed in pharmaMRI, with a focus on blood‐oxygen‐level‐dependent (BOLD) fMRI, arterial spin labeling (ASL), and cerebral blood volume (CBV) imaging. We then explore a range of experimental designs, highlighting key considerations for interpretability, reproducibility, and robustness. Subsequently, we review the analytical approaches used to extract meaningful drug‐related effects, from conventional region‐of‐interest (ROI) and connectivity‐based analyses to emerging methods such as receptor‐informed analysis and pharmacokinetic/pharmacodynamic (PK/PD) modeling. The review also discusses how integrating pharmaMRI with PET or electrophysiology enables richer, multimodal insights into molecular target engagement, drug‐induced neural activity, and hemodynamic responses. We then examine mechanistic findings from preclinical animal studies and discuss their translation into human research, including investigations in healthy volunteers and clinical populations. These studies have begun to identify potential biomarkers for treatment response, pointing toward future applications in personalized psychiatry. Finally, we consider how open science practices, harmonized protocols, and shared datasets can accelerate progress in the field. By synthesizing two decades of technical and conceptual development, this review aims to chart a roadmap for leveraging pharmaMRI to increase our understanding of psychiatric drug mechanisms and advance precision medicine in mental health.

## 2 Principles of Functional Imaging for PharmaMRI

**FIGURE 1:**
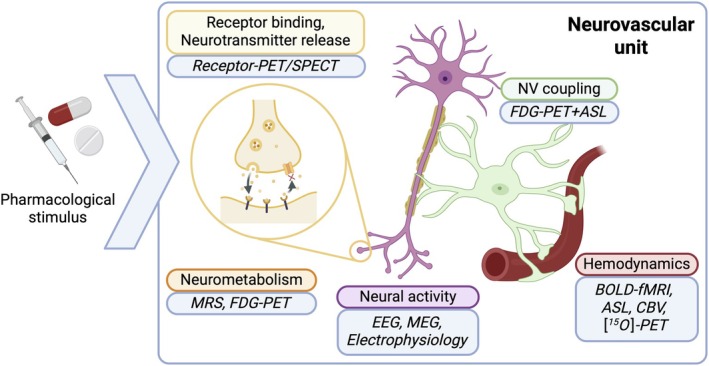
Principles of pharmaMRI. Different methods can be used to capture distinct aspects of drug‐induced changes to the neurovascular unit. Receptor‐specific PET and SPECT probes can capture target engagement or neurotransmitter release; Magnetic Resonance Spectroscopy (MRS) and FDG‐PET can assess neurometabolism; Electroencephalography (EEG), Magnetoencephalography (MEG), and electrophysiology can assess neural activity; a combination of FDG‐PET (to measure cerebral metabolic rate of glucose, CMR_Glu_) and specific ASL sequences (to measure cerebral metabolic rate of oxygen, CMR_O2_) can provide an index of neurovascular (NV) coupling; and finally, the hemodynamic response can be captured using BOLD‐fMRI, ASL, CBV, or [^15^O]‐tracer PET imaging.

PharmaMRI captures how pharmacological challenges influence brain function by measuring the cascade from receptor binding to hemodynamic responses (Figure 1). When a drug binds to its target receptor, it initiates intracellular signaling cascades that modulate neuronal firing rates. In turn, changes in neuronal activity drive local alterations in cerebral blood flow (CBF), volume (CBV), and oxygenation, which are the basis of ASL, CBV, and BOLD signals, respectively (Box 1). Thus, pharmaMRI provides a noninvasive readout of target engagement and downstream physiological effects. Importantly, pharmaMRI signals may also reflect direct vascular effects, independent of neuronal activity, via receptor subtypes expressed on microvascular smooth muscle cells (e.g., dopamine D1/D5) or astrocytes (e.g., D3) [3]. Other neuromodulatory receptors and factors can likewise influence astrocytic function and local vasodilation or constriction, thereby directly altering CBV or CBF [4, 5].

BOX 1 **Functional imaging techniques used in pharmaMRI.**

PharmaMRI relies on functional imaging techniques that capture hemodynamic responses to drug challenges. These methods reflect different aspects of cerebral physiology and are foundational to interpreting drug‐induced brain changes.

*BOLD fMRI*—The BOLD signal is the most widely used method in pharmaMRI. It indirectly reflects neural activity by measuring changes in the ratio of oxygenated to deoxygenated hemoglobin. Neural activation increases local metabolic demand, prompting an overcompensatory rise in CBF, which in turn alters local magnetic properties and produces a detectable BOLD signal. However, because BOLD is influenced by multiple physiological processes, including blood flow, blood volume, and oxygen metabolism, it offers a composite, indirect marker of neural activity and is sensitive to pharmacological modulation of vascular tone or neurovascular coupling.

*rCBV fMRI—*rCBV‐based imaging provides a more direct measure of cerebral blood volume changes within a brain region. rCBV‐sensitive pharmaMRI requires contrast agents (e.g., iron oxide nanoparticles) that remain within the vasculature, enhancing sensitivity to small vessels [6]. Therefore, it is also referred to as the “IRON” fMRI method, with the acronym denoting “increased relaxation for optimized neuroimaging”. This approach is especially common in preclinical studies and offers improved spatial specificity compared to BOLD. In pharmaMRI, rCBV can provide complementary insights into drug effects on vascular dynamics and capillary‐level responses.

*ASL MRI*—ASL is a noninvasive technique that quantifies absolute regional CBF by magnetically labeling inflowing arterial blood as an endogenous tracer. Unlike BOLD, ASL provides quantitative and regionally specific perfusion maps, making it particularly useful for studying baseline drug effects and slow‐onset pharmacodynamics (e.g., after oral drug administration). Compared to BOLD, ASL suffers from lower temporal resolution but offers superior reproducibility and physiological interpretability for pharmacological studies [7, 8].

Importantly, receptor subtype and drug mechanism determine the direction and magnitude of the hemodynamic response [2]. For example, dopamine D1 receptor agonists increase striatal BOLD and relative CBV (rCBV) signals, likely via vasodilatory pathways and increased excitatory tone. In contrast, dopamine transport blockers may reduce BOLD signals in basal ganglia regions by modulating tonic inhibitory control [9, 10]. Similarly, serotonin receptor subtypes (e.g., 5‐HT1A vs. 5‐HT2A) can produce opposing vascular responses depending on their excitatory or inhibitory downstream signaling [11]. The amplitude of the pharmaMRI signal often correlates with receptor occupancy for classic agonists or antagonists, enabling dose–response profiling [2, 12].

Interpreting pharmaMRI data requires careful attention to factors that can decouple neural activity from hemodynamic signals. Drugs may directly affect vascular tone (e.g., anesthetics or caffeine), alter global perfusion, or induce systemic changes in heart rate, respiration, or blood pressure [2]. Additionally, many psychiatric drugs act on multiple receptor types (“dirty drugs”), complicating signal attribution. Spatially, rCBV and ASL imaging may offer greater localization to capillary‐level changes than BOLD [13].

## 3 Study Designs and Experimental Considerations

### 3.1 Experimental Considerations

The timing of drug administration is determined by its pharmacokinetics, with pharmacological MRI during an acute challenge (often referred to as phMRI [14]) used for fast‐acting drugs. For phMRI, a drug can be administered through a route allowing rapid absorption and distribution (e.g., intravenous [*iv*], subcutaneous [*sc*], intranasal [*in*]), with the local changes in signal amplitude being the outcome measure [15, 16]. The reason for this method being typically applied to fast‐acting drugs is limitations in power to detect low‐frequency changes with BOLD fMRI and impracticalities of longer scans (e.g., for oral drugs, patches). As the drug engages with its target neurotransmitter receptors, it induces neuronal (de)activation, which in turn leads to a hemodynamic response through neurovascular coupling (Figure 1). If the post‐administration pharmacokinetics are stable for tens of minutes or longer, then pre‐post drug brain connectivity can also be analyzed [17, 18, 19], and additional assessments such as quantitative perfusion or magnetic resonance spectroscopy (MRS) can be added as required. phMRI can therefore offer insights into the functional effects and their distribution for specific drugs, and can be applied similarly in human and experimental animal studies, making it particularly well‐suited for translational drug discovery research [20]. Notably, intravenous drugs can also induce significant cardiovascular effects, which can complicate the interpretation of neural versus vascular contributions to the MRI signal. Concurrent monitoring of heart rate, blood pressure, and peripheral oxygenation can be used to evaluate these effects and used to model physiological noise during data analysis to help dissociate drug‐induced neural activity from systemic hemodynamic confounds. In animal studies, in addition to *iv* administration, drugs are often administered intraperitoneally (*ip*), which provides quick systemic uptake with potentially fewer direct vascular confounds than intravenous routes.

### 3.2 Study Designs

**FIGURE 2:**
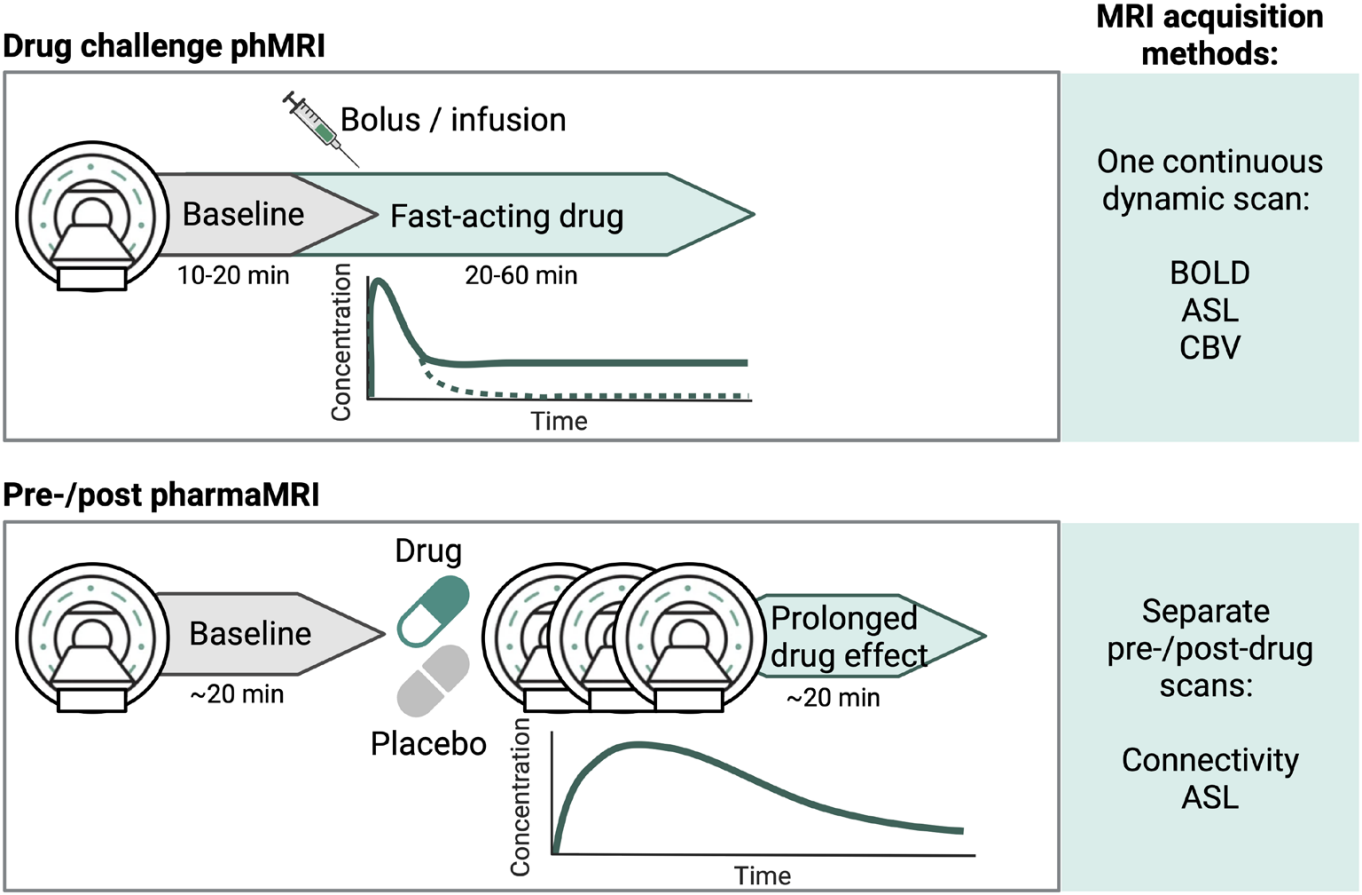
Study designs for pharmaMRI. Top panel: *Drug challenge phMRI* A baseline MRI scan is conducted for 10–20 min, followed by the administration of a fast‐acting drug. The MRI scan continues dynamically for 20–60 min post‐administration. This approach is suitable for fast‐acting drugs and utilizes continuous dynamic imaging methods such as Blood Oxygen Level‐Dependent (BOLD), Arterial Spin Labeling (ASL), or Cerebral Blood Volume (CBV) MRI to capture acute changes in signal amplitude over time. Bottom panel: *Pre‐/post pharmaMRI* A baseline MRI scan of approximately 20 min is performed, followed by drug or placebo administration. After a period allowing for drug distribution, a second MRI scan is conducted to assess prolonged drug effects. This design employs separate pre‐ and post‐drug scans, focusing on functional connectivity analyses and ASL to evaluate sustained changes in brain function.

**FIGURE 3:**
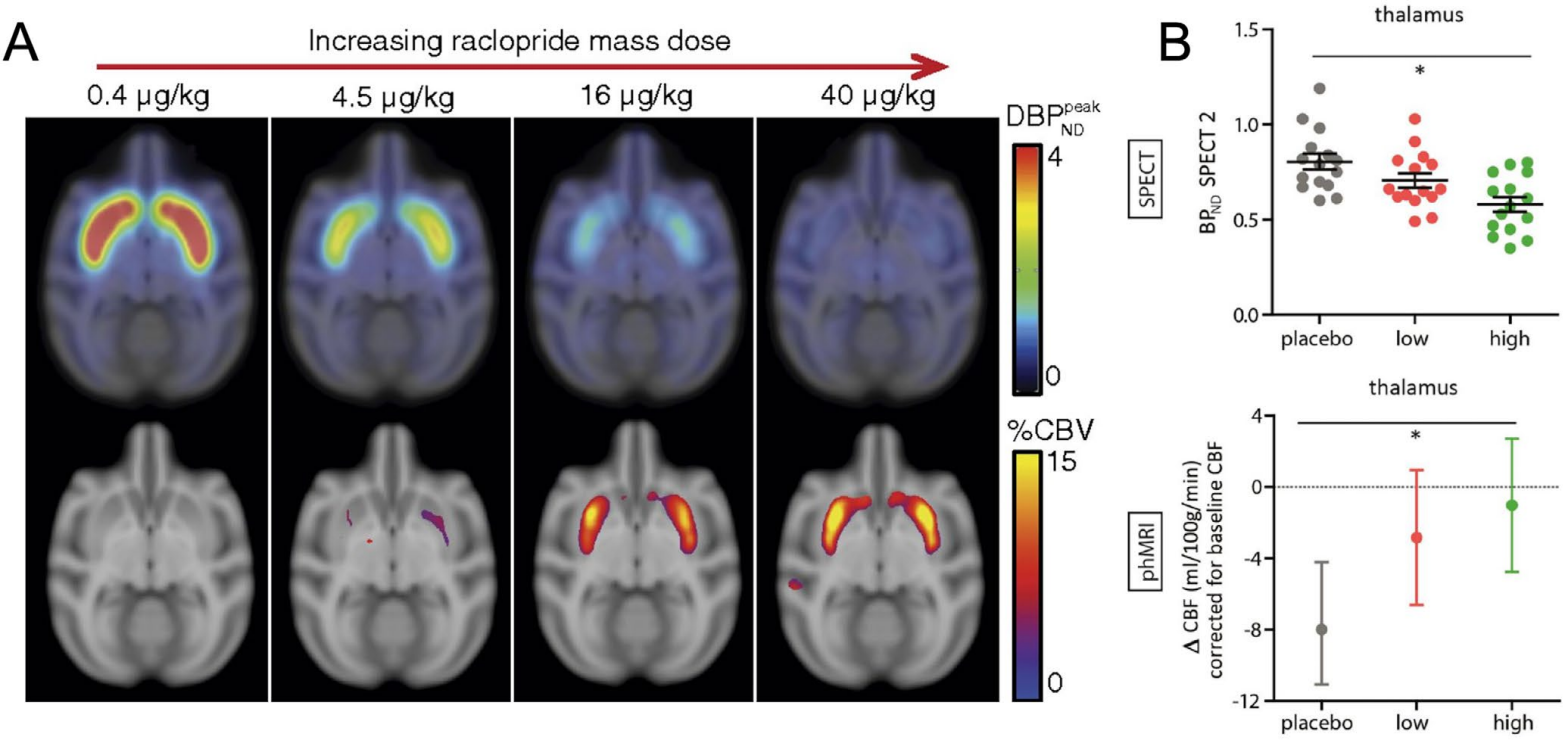
Dose‐dependent pharmaMRI effects. (A) Dopamine D2/D3 receptor‐specific PET data showing increasing [11C]raclopride displacement, and fMRI data showing increased CBV in the striatum with increasing pharmacological doses of the D2/D3 receptor antagonist raclopride, in a nonhuman primate model [21]. (B) SPECT data showing lower [123I]FP‐CIT BP, and fMRI/ASL data showing lower response to a subsequent intravenous citalopram dose with increasing pre‐treatment with an oral citalopram dose, in healthy volunteers [22].

PharmaMRI studies typically begin with a baseline MRI scan, followed by drug administration and a subsequent scan (pre‐post design), or, in some cases, continuous imaging during drug infusion (Figure 2). Additionally, placebo controls can be used, for example, by administering saline on a different day in the same subjects. While most human studies employ a single administration pre‐post design, we can distinguish a number of other study designs. Dose–response paradigms are a powerful approach, enabling researchers to correlate drug dose with receptor occupancy and functional brain changes. For example, increasing doses of the dopamine D2 antagonist raclopride have been shown to correlate with occupancy and increasing striatal CBV changes [21] (Figure 3A). Ren et al. [12] found that increasing doses of D‐amphetamine caused dose‐dependent dopamine release and cAMP activity, while pharmaMRI showed that low doses decreased striatal CBV, and high doses increased it, possibly reflecting a shift from D2/D3 to D1/D5 receptor dominance. In humans, citalopram was shown to modulate thalamic pharmaMRI responses in a dose‐dependent manner, with higher oral doses attenuating the CBF decrease typically seen after an intravenous challenge [22] (Figure 3B). Similarly, S‐ketamine elicited robust, dose‐dependent increases in pharmaMRI signal in the dorsal‐frontal, cingulate, and insular cortices, which correlated with both subjective dissociation and opioid and glutamate receptor distribution maps [23].

**FIGURE 4:**
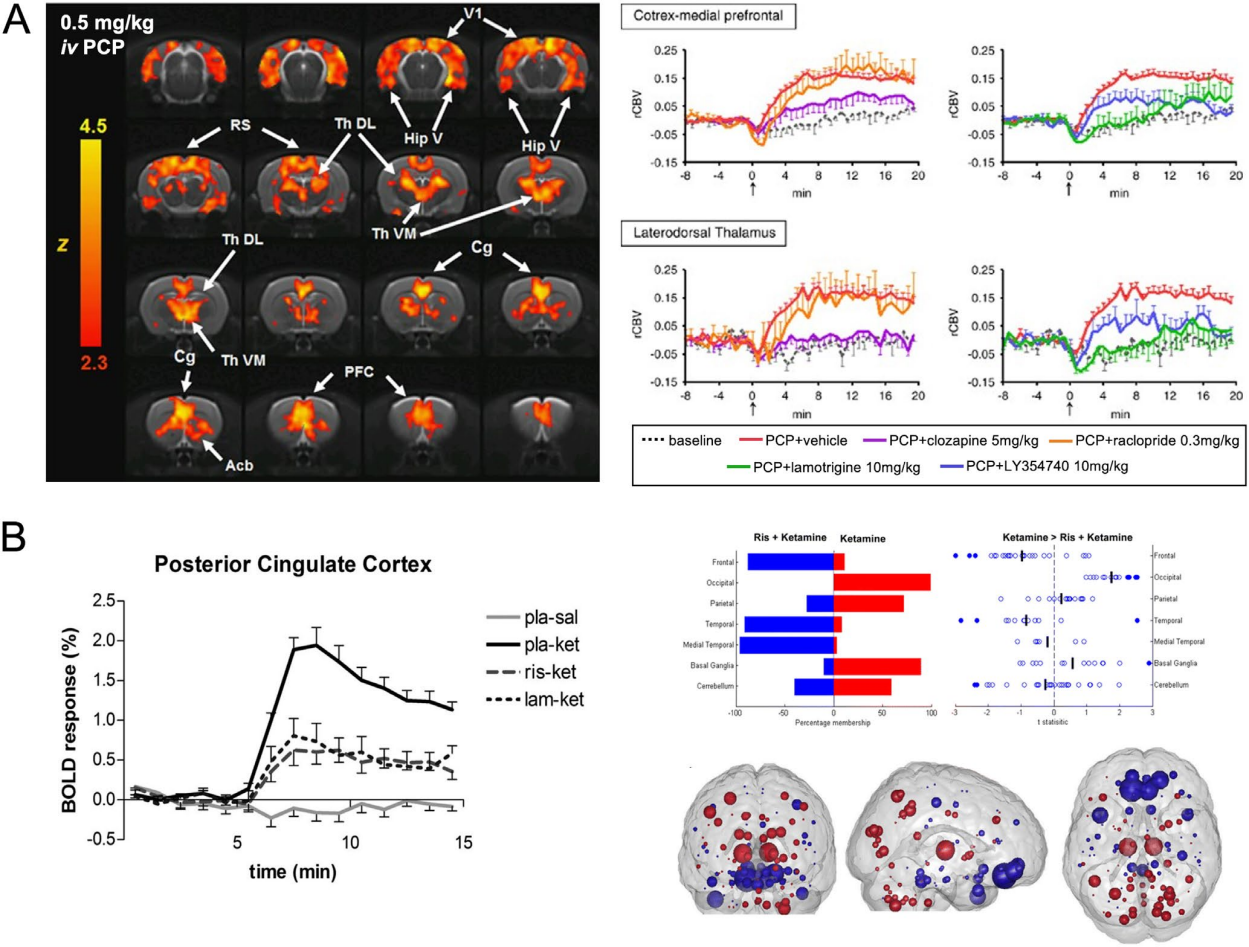
Receptor blocking pharmaMRI studies in dissociative drugs. (A) Preclinical study in rats showing that pre‐treatment with clozapine, lamotrigine, and a mGluR2/3 agonist blocked the change in rCBV induced by phencyclidine (PCP), whereas raclopride did not. Adapted from [20]. (B) Human study demonstrating that pre‐treatment with risperidone blocked both ketamine‐induced changes in activity and connectivity, whereas lamotrigine only blocked the activity but not the connectivity response. Adapted from [24] and [17].

Receptor‐blocking pretreatment studies (Figure 4) represent another important design, in which a blocking agent is administered beforehand to attenuate the response to the drug under investigation, therefore helping to isolate target‐specific drug effects. For example, pretreatment with a selective 5‐HT2C antagonist largely reversed mCPP‐induced BOLD signal changes in limbic and basal ganglia regions, while a 5‐HT1B antagonist had minimal impact. Together, this demonstrates that mCPP's effects on feeding behavior and brain activation are mediated predominantly via 5‐HT2C receptors [25]. Ketamine‐induced changes in hemodynamic functional measures are attenuated by lamotrigine in both animals [20] and humans [24, 26], indicating the role of glutamate release in mediating the changes in signal amplitude, but not connectivity [17]. Risperidone also attenuated the signal amplitude and connectivity after ketamine [17, 24], suggesting that both glutamatergic and monoaminergic modulation are important in shaping the brain's hemodynamic response to NMDA receptor antagonism. This technique can be applied to novel compounds to validate mechanisms and determine dose‐related effects [27].

### 3.3 Experimental and Design Considerations in Clinical phMRI

Conducting phMRI studies in clinical populations requires attention to both technical, patient, and drug‐related factors. phMRI in clinical populations can generally be performed on widely available 1.5 T and 3 T MRI systems. Most modern scanners are equipped with multi‐channel phased‐array head coils, which provide sufficient sensitivity for BOLD and ASL measurements without the need for highly specialized hardware. While ultra‐high‐field systems (7 T and above) can improve signal‐to‐noise ratio and vascular specificity, their use remains limited to specialized research centers and is not essential for most clinical phMRI applications. Patient‐related factors play an equally important role. Shorter acquisition times and motion correction strategies are particularly important in psychiatric populations, where patient comfort and adherence to the protocol are critical. Patient sedation should be avoided wherever possible, as it can directly modulate neurovascular coupling [28]; when necessary, its effects on the imaging signal should be explicitly acknowledged. Drug‐related considerations include prior medication exposure versus drug‐naïve status, which can substantially alter baseline neurotransmission and receptor availability and thus influence phMRI responses. Similarly, age may impact vascular reactivity and neurochemical sensitivity and should be considered when interpreting developmental or aging cohorts [29].

### 3.4 Design Considerations for Preclinical Studies

Specialized hardware is crucial for successful animal pharmaMRI. This includes the need for animal scanners and/or small‐diameter, high‐sensitivity radiofrequency coils that are optimized to the animal anatomy [30]. These coils are ideally integrated into animal holders that provide consistent support and minimize motion. Utilizing higher magnetic fields (e.g., 7 T, 9.4 T, or even 15 T) enables shorter imaging times while significantly enhancing spatial resolution, allowing more precise anatomical and functional mapping. Additionally, employing high gradient strengths and rapid slew rates is vital for capturing dynamic brain activity, particularly in small animal brains, where spatio‐temporal resolution demands are high.

Injectable iron oxide nanoparticles (e.g., ferumoxytol, monocrystalline iron oxide nanoparticles [MION]) are preferred for animal pharmaMRI due to their enhanced sensitivity compared to endogenous BOLD contrast. Outcome measures reflect rCBV changes (see Box 1). Iron oxide nanoparticles shorten the T2* relaxation time, thereby boosting contrast‐to‐noise ratios by approximately 3‐fold compared to BOLD signals at 3 T [31]. The method has demonstrated advantages over traditional BOLD imaging, including higher detection power, reduced susceptibility artifacts, and fewer confounding physiological factors [6]. For example, clear regional brain activation using MION‐based CBV imaging in response to amphetamine was shown in a number of regions not described previously [32]. Iron oxide nanoparticles are typically administered at the beginning of an imaging session, and their prolonged half‐life ensures stable contrast over several hours. Importantly, the interpretation of data from the IRON pharmaMRI experiment remains straightforward, and CBV‐based outcome measures can be reliably compared with those obtained using traditional BOLD contrast [31].

**FIGURE 5:**
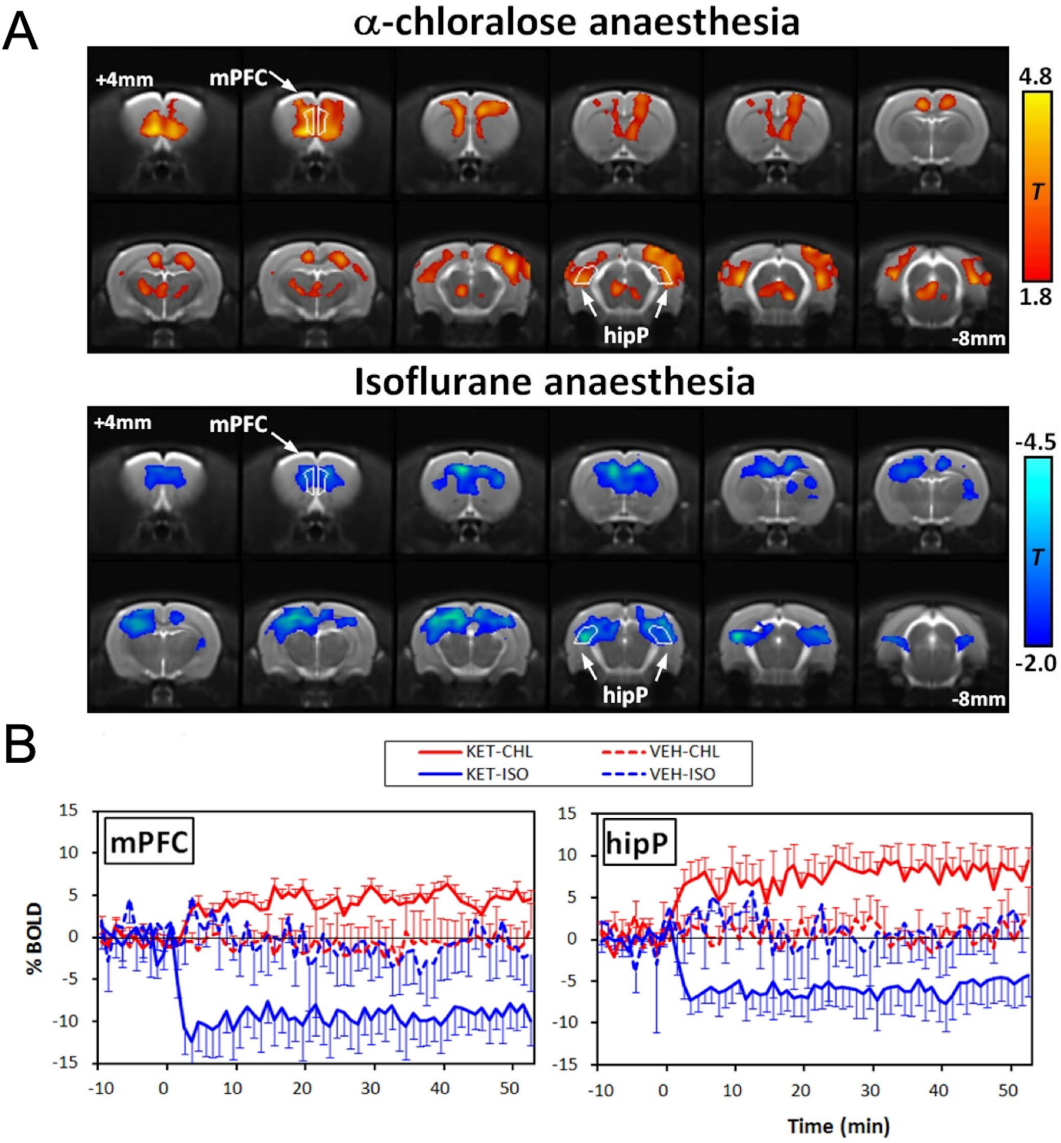
Differential effects of anesthesia on ketamine response in rodents. (A) Under alpha‐chloralose (CHL), ketamine (KET) administration induces increases in BOLD‐fMRI signal, while under isoflurane (ISO) decreases occur in similar areas. (B) %BOLD timecourses in two exemplary brain areas. VEH = vehicle; mPFC = medial prefrontal cortex; hipP = posterior hippocampus. Adapted from [35].

Most animal imaging studies are performed in anesthetized conditions. Although more convenient, and often the only practically available approach, anesthesia is one of the major discussion points across the community [33]. Some choices of anesthesia have been shown to be inadequate, such as isoflurane at higher doses (> 1%) in rodents, while the combination of dexmedetomidine and low‐dose isoflurane has been recommended as being one of the most suitable, due to its minimal cardiovascular and neurovascular effects, especially in the context of preserving resting‐state architecture [34]. Yet, light sedation as provided by dexmedetomidine + isoflurane might not be sufficient for certain pharmacological interventions, so that this balance needs to be carefully evaluated for each study design. Moreover, interactions between the compound and anesthetic need to be considered, as these cannot be corrected for using post‐processing methods. For instance, volatile anesthetics can alter the directionality of changes elicited by NMDA antagonists, as demonstrated with the combination of ketamine and isoflurane (Figure 5) [35], as well as PCP and halothane [36]. One way to circumvent the issue is awake imaging [37], which, however, also comes with its own caveats: animals need to be trained over several weeks for compliance, and stress needs to be closely monitored using techniques such as pupillometry. Still, awake studies provide important data for validation and enable a translational pathway to human studies. Awake pharmaMRI studies in rodents and nonhuman primates have successfully characterized neural responses to, for example, psychostimulants, and have underscored the value of this approach for bridging preclinical and clinical research [31, 38].

### 3.5 Design Considerations for Human Studies

In human studies, pharmaMRI can be performed at rest or during specific tasks. Resting‐state pharmaMRI captures drug engagement with its target, whereby it induces neuronal (de)activation, which in turn leads to a hemodynamic response through neurovascular coupling. This can therefore offer insights into the distribution and function of specific neurotransmitter systems (or other targets), making it particularly well suited for drug discovery studies in which outcomes are defined either by expected changes in activity or connectivity within specific brain systems, or through data‐driven descriptions of such changes. Notably, resting‐state pharmaMRI can refer both to approaches that measure direct changes in the amplitude of hemodynamic signals following intravenous drug administration, and to studies that examine alterations in functional connectivity before and after drug exposure. Conversely, task‐based pharmaMRI measures drug‐induced changes in brain activity during cognitive, emotional, or sensory tasks. For example, acute intravenous citalopram significantly increased bilateral amygdala reactivity to fearful faces, an effect that was positively associated with presynaptic 5‐HT1A receptor availability in the dorsal raphe nucleus, suggesting that early SSRI effects on emotional processing are modulated by serotonin autoreceptors [39]. This approach is ideal for probing targeted neurotransmitter systems, such as dopamine system changes during reward processing, but is influenced by performance variability, and the interaction effects of the drug and task are difficult to disentangle.

## 4 Analysis Methods

Depending on the experimental design and acquisition choices, a multitude of pharmaMRI analysis approaches have been developed and applied. These can broadly be divided into methods directly analyzing drug‐induced changes in signal intensity (activity‐based analysis) and indirect methods investigating drug‐modulated changes in functional connectivity between regions (connectivity‐based analysis).

### 4.1 Activity‐Based Analysis

Activity‐based pharmaMRI analyses focus on detecting signal changes over time following drug administration. A common approach in BOLD fMRI is time‐series analysis using the general linear model (GLM), which tests whether the observed fMRI signal at each voxel or within regions of interest (ROIs) fits a predicted model based on the expected pharmacodynamic response. ROIs can be anatomically defined, literature‐based, or derived from independent functional studies. In this framework, the GLM models the fMRI signal as a linear combination of temporal regressors representing hypothesized phases of the drug response.

One of the simplest approaches uses a boxcar regressor to divide the scan into pre‐ and post‐infusion blocks, allowing for a basic contrast of drug effects but lacking sensitivity to gradual signal changes. On the other end of the spectrum, modeling each post‐infusion time point separately increases sensitivity while inflating the risk of false positives due to the greater number of statistical tests and multiple comparisons. An intermediate approach is the pseudo‐block design, where the pre‐infusion period serves as a baseline and the post‐infusion scan is segmented into a series of discrete blocks, each analyzed as a separate test condition [26]. This model allows for the detection of temporal dynamics while maintaining a manageable model complexity and controlling for multiple comparisons. To better reflect the biological processes underlying drug effects, waveform‐based regressors have also been introduced. These use gamma‐variate or other analytically defined functions to approximate drug uptake, binding, and wash‐out, either empirically derived or informed by pharmacokinetic/pharmacodynamic (PK/PD) models (e.g., [16, 40]). Wavelet‐based methods have also been applied to capture more complex or nonlinear response patterns [41].

For ASL‐based pharmaMRI, particularly when acquired across separate sessions, activity‐based analyses typically involve calculating changes in absolute or relative CBF from quantitative perfusion maps. Unlike BOLD or rCBV‐based IRON imaging, ASL yields quantitative data suitable for between‐session comparisons.

### 4.2 Connectivity‐Based Analysis

Connectivity‐based pharmaMRI analyses assess how pharmacological agents modulate interactions between brain regions. A common approach is seed‐based analysis, which quantifies the temporal correlation between a predefined region (the seed) and all other voxels in the brain. This method is hypothesis‐driven and suited to detect both expected and unforeseen connectivity changes induced by drug administration. ROI‐to‐ROI analysis, in contrast, focuses on interactions between multiple predefined regions, typically defined using anatomical or functional atlases. When constrained to specific neural circuits, this approach can test targeted hypotheses about drug effects on systems such as the corticostriatal or limbic circuits. When applied at the whole‐brain level, it enables the construction of connectivity matrices, from which graph‐theoretical measures can be extracted to describe global or local network organization. Independent Component Analysis (ICA), a data‐driven method decomposing fMRI data into spatially independent networks, is another method that has been successfully applied in pharmaMRI to isolate networks sensitive to drug effects, for example, detecting CBF fluctuations related to amphetamine infusion [42].

**FIGURE 6:**
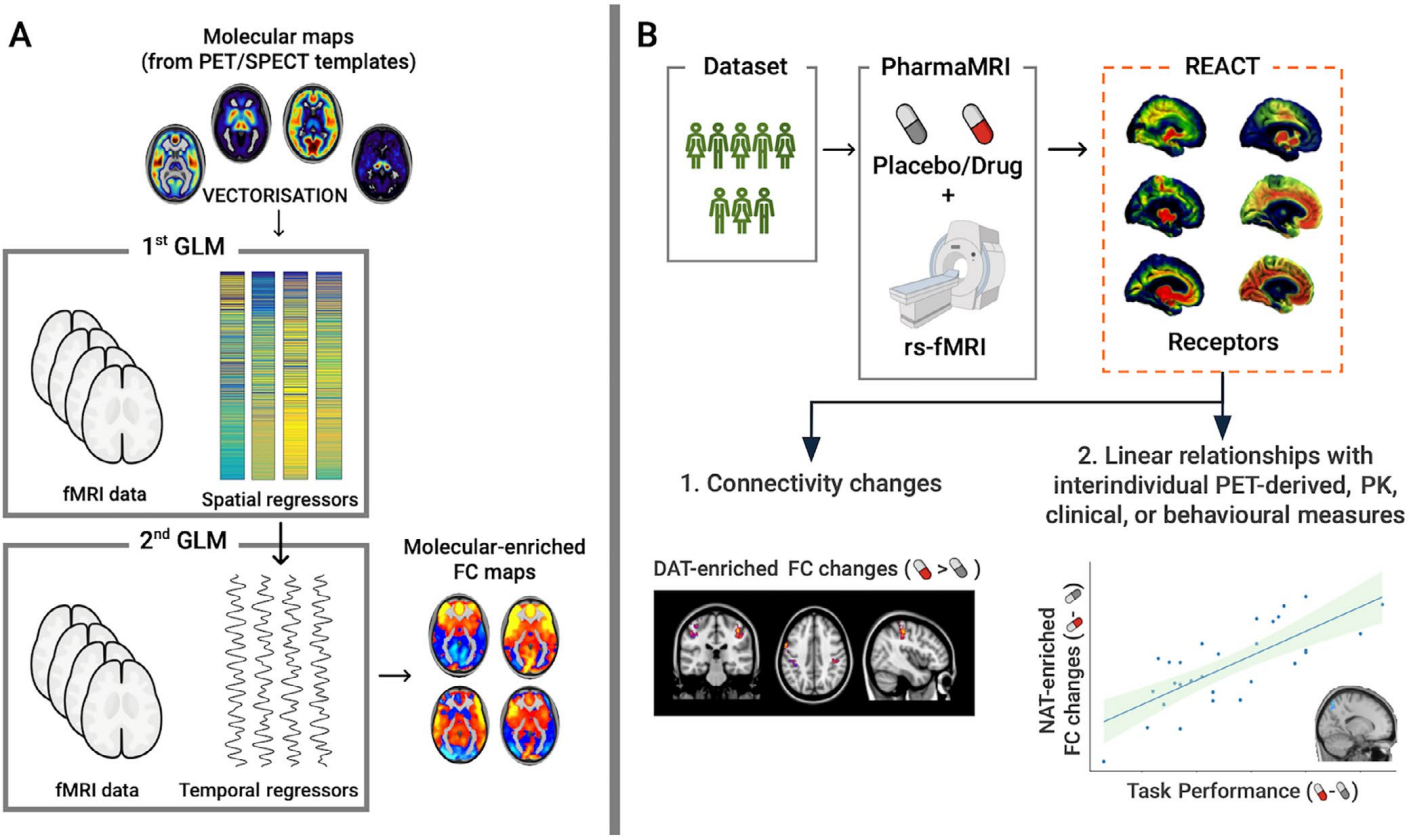
Overview of the REACT framework and its application to a pharmacological fMRI study. (A) The REACT method integrates molecular information from PET or SPECT‐derived receptor density maps into a two‐step regression analysis of fMRI data. In the first GLM, molecular templates are vectorized and used as spatial regressors to extract dominant time series weighted by receptor distribution. In the second GLM, these time series are used as temporal regressors to generate molecular‐enriched functional connectivity (FC) maps. (B) Example application in a placebo‐controlled resting‐state fMRI study of methylphenidate. The REACT framework was applied using molecular templates for dopaminergic and noradrenergic targets. Resulting analyses revealed (1) Methylphenidate‐induced changes in DAT‐enriched FC, and (2) significant linear relationships between individual differences in NAT‐enriched FC and task performance (adapted from [44]).

A key limitation of the previous approaches is that they do not incorporate information about the molecular architecture of the brain, nor do they account for how molecular systems modulate functional connectivity during pharmacological challenges. To address this gap, novel methods have been developed in recent years to enrich functional MRI data with molecular information. One of these techniques is Receptor‐Enriched Analysis of functional Connectivity by Targets (REACT) [43] (Figure 6). REACT integrates molecular receptor density maps, derived from PET or SPECT studies, into a two‐step linear regression framework. First, receptor distribution templates (e.g., for serotonin or dopamine transporters) are used as spatial regressors in a first GLM to extract dominant time series from fMRI data. These time series, weighted toward regions of high receptor density, are then used in a second GLM to identify brain areas whose dynamics covary with the molecular‐enriched signal. The resulting maps offer a biologically grounded view of functional connectivity, linking network changes more directly to the underlying molecular systems engaged by the pharmacological agent.

In practice, the choice between analyses depends not only on design and hypotheses but also on statistical power, multiple comparisons, and reproducibility across sites. ROI‐based approaches provide high sensitivity for targeted hypotheses, whereas connectivity‐based and data‐driven methods (e.g., ICA, REACT) allow systems‐level insights but demand larger samples and stricter control of false positives. Each method also introduces model‐specific assumptions, such as the form of GLM regressors that can bias results; these can be mitigated by cross‐validation, replication in independent datasets, incorporation of physiological or pharmacokinetic covariates, and transparent reporting of analytic choices.

## 5 Multimodal Imaging

The interpretative power of pharmaMRI can be significantly enhanced when integrated with other modalities, such as microdialysis, PET, or electrophysiology. Multimodal imaging allows simultaneous characterization of neural responses and underlying molecular changes, thus enabling a deeper understanding of pharmacological mechanisms. Recent technological advances, particularly the emergence and development of simultaneous PET/MRI systems, have transformed this field by providing concurrent molecular and functional data. This integration offers unique opportunities to bring together expertise from traditionally different research fields and to advance the interpretation of complex pharmacological interventions.

### 5.1 Neurochemical Techniques

Early preclinical studies critically advanced the field of pharmaMRI by pairing it with neurochemical techniques such as microdialysis. This provided essential evidence that acute drug‐induced changes detected by pharmaMRI reflect genuine neuronal activity, rather than vascular or nonspecific physiological effects. Microdialysis samples extracellular fluid to quantify neurotransmitter release over slower timescales. Although it has lower temporal resolution compared to voltammetry (typically minutes), microdialysis enables quantitative assessment of a wide range of neurochemicals over time. Seminal research has demonstrated that changes in BOLD activity after systemic drug administration closely parallel measured increases in extracellular levels of target neurotransmitters [12, 45, 46], thereby reinforcing the interpretation that pharmaMRI responses can reliably index drug‐induced neurochemical events. Another neurochemical method that may be of interest to use in conjunction with pharmaMRI is voltammetry, which measures rapid fluctuations in neurotransmitter concentrations, such as dopamine or serotonin, via electrochemical changes at a microelectrode [47]. Magnetic resonance spectroscopy (MRS) offers a noninvasive complementary approach by enabling in vivo quantification of neurochemicals such as glutamate, GABA, and N‐acetylaspartate. Unlike fMRI, which provides indirect measures of neuronal activity via hemodynamic coupling, MRS can directly assess neurochemical changes induced by pharmacological interventions. This makes it particularly valuable for probing target engagement and drug mechanisms at the molecular level [48]. Proton MRS (^1^H‐MRS) has been widely applied in psychiatry, for example, to study ketamine‐induced glutamate changes in [49]. More recently, advanced techniques such as edited MRS have enabled more reliable quantification of low‐concentration metabolites like GABA. Phosphorus MRS (^31^P‐MRS) has been used in a limited number of drug studies to assess treatment effects, for example, investigating changes in energy metabolism metrics under antipsychotic [50] and lithium treatment [51, 52].

### 5.2 Molecular Insights From PET/MRI

PET offers unprecedented specificity and sensitivity for imaging distinct molecular targets in vivo. Molecular PET targets typically include enzymes, neurotransmitter receptors, or transporters, with new radiotracers continuously developed to probe novel biological pathways relevant to psychiatry.

Historically, PET using [^18^F]fluorodeoxyglucose (FDG‐PET), a radiolabeled glucose analogue, served as the main tool for functional brain imaging prior to the widespread adoption of fMRI [53]. fMRI has since become the dominant method for imaging brain function due to its ease of access, nonionizing radiation, and superior spatiotemporal resolution. However, more recent technological and methodological developments have sparked renewed interest in pharmacological FDG‐PET. First, early studies relied on two static PET scans (placebo and drug) acquired after separate bolus injections [53]. The advent of functional PET (fPET) using continuous FDG tracer infusion [54] enables (i) dynamic tracking of glucose utilization within a single session, (ii) temporal resolutions approaching fMRI studies [55], and (iii) the incorporation of simple or complex task paradigms [54, 55]. Although BOLD fMRI also supports such designs, FDG‐fPET may be less susceptible to certain confounds, such as peripheral drug effects.

Beyond FDG, a variety of receptor‐specific radiotracers have provided important molecular insights complementary to pharmaMRI in psychiatry. Key neurotransmitter systems targeted include dopamine, serotonin, glutamate, noradrenaline, and opioids, among others [56]. PET remains the gold standard for determining receptor occupancy in vivo and for defining therapeutic windows, particularly for antipsychotic drugs. For instance, occupancy of dopamine D2/D3 receptors by antipsychotics, like risperidone or aripiprazole, has been robustly demonstrated [57]. Additionally, the potential effects of inflammation on psychiatric disorders and pharmacological interventions have also been explored using PET radiotracers targeting the translocator protein (TSPO) as a marker for activated microglia [58].

The emergence of simultaneous PET/fMRI has fueled studies yielding complementary insights, especially in pharmacology. For instance, the administration of a vasoactive compound can induce divergent responses in fPET and fMRI [59], likely due to peripheral vascular confounds affecting only the latter. Beyond pharmacology, fPET‐fMRI has advanced the understanding of fundamental brain processes, such as default‐mode network deactivation during tasks [60]. Furthermore, receptor‐specific tracers used in simultaneous PET/fMRI studies have significantly advanced our understanding of drug mechanisms at both molecular and system levels (Figure 3A). An early demonstration of neurovascular coupling linked to receptor occupancy involved simultaneous PET/fMRI experiments with dopamine D2/D3 receptor antagonists, mapping spatial, temporal, and dose‐dependent relationships between receptor occupancy and hemodynamic responses [21]. Similar relationships showing neurovascular coupling to receptor occupancy have since been demonstrated, for example, for serotonin and opioid systems [1, 61].

Understanding the biological complexity underlying imaging signals is critical, as each neurotransmitter system and pharmacological agent has unique properties affecting PET and pharmaMRI signals in varying ways. Receptor internalization after agonist exposure exemplifies such complexity: agonist‐induced internalization of G protein‐coupled receptors may prolong the PET occupancy timeline due to changes in postsynaptic receptor density, while simultaneously shortening the pharmaMRI signal by desensitizing downstream signaling pathways [62]. Simultaneous PET in combination with pharmaMRI is crucial for investigating these types of time‐sensitive biological processes.

Finally, recognizing the strengths and limitations of each imaging modality is essential for multimodal imaging. PET excels in detecting changes that align with the specific radiotracer kinetics, which can range from minutes to hours. At the same time, it can reliably confirm target engagement. Conversely, pharmaMRI requires robust baseline measurements and careful statistical handling due to inherent noise and variability. While small drug‐induced changes detectable by PET receptor occupancy may be more challenging to quantify consistently using pharmaMRI alone, pharmaMRI is uniquely able to capture downstream or network‐level functional effects of drug action that may not be directly observable with PET, providing complementary systems‐level insights [1, 63].

The emergence of total‐body PET is a recent breakthrough that may fuel insights for pharmaMRI. These systems offer unprecedented sensitivity and temporal resolution, potentially capturing second‐by‐second molecular dynamics across the entire body at lower PET tracer doses [64, 65]. With respect to pharmacology, they may enable tracking of acute effects of drugs on additional physiological levels, with spatiotemporal resolution approaching that of pharmaMRI. They also have the potential for prolonged exposure studies that can track pharmacological interventions on longer timescales.

### 5.3 Electrophysiological Correlates

Pharmacological neuroimaging is greatly enhanced by integrating electrophysiological methods, as these offer direct, real‐time measures of neural activity. Among these, electroencephalography (EEG) is the only method that has been simultaneously combined with fMRI in pharmacological studies. Simultaneous EEG‐fMRI combines EEG's millisecond temporal precision with fMRI's whole‐brain coverage, enabling dissociation of neuronal from vascular drug effects and the identification of frequency‐specific signatures of drug action.

Studies in healthy volunteers and patients with major depressive disorder (MDD) have examined ketamine infusion using EEG‐informed phMRI analyses [66, 67]. In both populations, EEG regressors based on specific frequency bands revealed distinct temporal profiles of neural responses that were not captured by a single pharmacokinetic response model. Importantly, no EEG time series correlated with the commonly reported BOLD decrease in the subgenual anterior cingulate cortex (sgACC), suggesting that this effect may largely reflect physiological noise, particularly cardiac pulsatility, rather than a direct neural response to ketamine. A task‐based placebo‐controlled crossover study [68] further demonstrated the value of EEG‐fMRI in pharmacological research. Comparing ketamine and midazolam during a working‐memory task, the authors found that ketamine attenuated both task‐related theta power increases and alpha power decreases and disrupted the normal positive coupling between frontal‐midline theta power and BOLD activity in working‐memory‐related regions. A similar approach was applied to psychedelics; a placebo‐controlled EEG‐fMRI study of intravenous *N*,*N*‐dimethyltryptamine (DMT) in healthy volunteers found widespread increases in global functional connectivity and altered oscillatory dynamics. EEG‐informed analyses consistently implicated changes along the brain's principal sensory‐association gradient, underscoring the value of EEG‐fMRI for linking neurophysiological and network‐level drug effects [69].

Although magnetoencephalography (MEG) cannot be recorded simultaneously with fMRI, it offers superior spatial localization of cortical oscillations compared to EEG, owing to its sensitivity to tangential currents and reduced susceptibility to signal distortion. Pharmacological MEG has proven valuable in characterizing drug‐induced changes in oscillatory activity, network synchrony, and connectivity [70]. Additionally, within‐subject multimodal designs, such as those investigating the effects of psychedelics [71], can provide meaningful mechanistic insights.

## 6 Mechanistic Insights From Preclinical Studies: Animal Models

Animal pharmaMRI studies have been pivotal in understanding the fMRI response and neural mechanisms underlying pharmacological stimulation. Foundational work by Chen et al. [45, 72] established the capability of pharmaMRI to visualize drug‐induced dopaminergic activity. This pioneered the use of pharmaMRI in addiction research. Subsequent studies highlighted neuroadaptive changes resulting from acute and chronic psychostimulant exposure [12, 73, 74, 75], providing crucial insights into addiction‐related circuitry and brain plasticity. Contributions from Jenkins et al. [2, 32] and Mandeville et al. [76] emphasized methodological rigor, highlighting the advantages of CBV‐based imaging and iron oxide contrasts. Further insight into CBV pharmaMRI sensitivity to modulation of dopamine and other neurotransmitters has been provided through a variety of pharmacological interventions [77, 78, 79].

Widespread brain network effects relevant to depression have been investigated using SSRIs as pharmacological stimuli in rodents: Klomp et al. [80] revealed how SSRIs modulate serotonergic pathways, while Grandjean et al. [81] provided a comprehensive map of serotonergic responses to acute stress and SSRI treatment in rodents. Additional insights into receptor subtype‐specific mechanisms and serotonergic network functionality have been gained from serotonergic modulation using pharmaMRI in nonhuman primates [61, 82].

PharmaMRI has also been employed to understand the effect of glutamatergic NMDA receptor antagonists, modulation by dopamine antagonists, and their parallels with clinical schizophrenia [36]. Understanding the role of glutamate and its modulation by dopamine and other neurotransmitter systems remains an important area of investigation.

Together, these studies form a comprehensive framework bridging molecular pharmacology and functional neuroimaging, laying critical groundwork for translational psychiatric research and therapeutic advancements.

### 6.1 Invasive Techniques: Optogenetics, Chemogenetics, and Genetic Manipulations

Among the most unique advantages of animal studies is the ability to perform targeted invasive manipulations. By specifically silencing or activating a certain population of neurons in a region, chemogenetic and optogenetic approaches can offer mechanistic insight to whole‐brain effects such as those elicited by drugs. For instance, optogenetic manipulations have reproduced the antidepressant effects of ketamine [83]. Using chemogenetics, it was possible to directly compare brain‐wide effects of direct activation of serotonergic neurons to those elicited by the systemic administration of selective serotonin reuptake inhibitors [84]. Going beyond pharmacological studies, chemogenetic and optogenetic approaches could offer important mechanistic evidence on the substrate of functional connectivity in different areas [85, 86], on neurochemical modulation of the default‐mode network [87], or on the nature of BOLD signal changes in the dopaminergic system [88, 89]. Therefore, also without being directly combined with pharmacology, such interventions will play an essential role in putting into context the signal changes elicited by compounds acting at different receptors and neurotransmitter systems. In the future, directly adding such approaches to observe the effects of targeted manipulation on the drug‐elicited fMRI responses will be a powerful tool to better understand pharmacological fMRI signals.

## 7 Clinical Studies

Building on findings from preclinical pharmaMRI, several psychoactive compounds have also been extensively studied in humans, providing insight into their systems‐level effects. Methylphenidate is a relatively well‐characterized example in ADHD. Clinical studies for insight into drug action can also include model systems, particularly well‐suited for translating findings from small animal fMRI. Here we use ketamine as a well‐characterized example (see Section 3 and Figure 4).

Methylphenidate is a psychostimulant best known for its use in treating attention deficit hyperactivity disorder (ADHD) and narcolepsy, with growing interest in its potential for addiction treatment. Its primary mechanism involves blocking the reuptake of dopamine and norepinephrine, thereby increasing their synaptic availability and enhancing neurotransmission [90]. Preclinical pharmaMRI studies have demonstrated that methylphenidate administration leads to increased activity in dopaminergic pathways, particularly within the striatum and prefrontal cortex, regions implicated in attention, reward, and executive function [91]. These studies have helped delineate the circuit‐level effects of methylphenidate. In clinical research, most human pharmaMRI studies with (oral) methylphenidate have focused on task‐based designs and connectivity analyses to determine the mechanisms of action in healthy volunteers and patient groups. Task‐based pharmaMRI studies often examine the drug's effects on cognitive control, attention, and reward processing [92], while connectivity‐based approaches explore how methylphenidate modulates functional networks, such as the default mode and fronto‐striatal circuits [93]. PharmaMRI has brought important insights into the mechanism of action of stimulants, such as normalization of the inferior frontal gyrus being important for inhibitory control in ADHD and similarly a restoration of the difference between the dorsal attention network and default mode network to reduce mind‐wandering and improve cognitive function. The relative importance of dopamine and norepinephrine transporter blockade across different cognitive functions remains poorly understood in both volunteer and patient studies, although REACT methodology is beginning to address this question [43, 94] (Figure 6B).

Ketamine is a complex drug studied via pharmaMRI for its roles in anesthesia, psychosis modeling, and as a rapid‐acting antidepressant. At low doses, it produces analgesic, dissociative, and antidepressant effects; at higher doses, it induces anesthesia [95]. Subanesthetic doses can mimic some symptoms of schizophrenia, making ketamine a common model for studying the disorder [96], in particular regarding the role of glutamate system modulation. Subanesthetic doses increase cortical glutamate release, as measured with MRS, which correlates with psychosis‐like experiences [23, 97] as well as increased default mode network signal during a working memory task, which correlates with negative symptoms [98]. Research shows that subanesthetic ketamine increases glutamate activity in the prefrontal cortex, likely by blocking presynaptic NMDA receptors and disinhibiting glutamatergic neurons via GABAergic interneurons [99]. In contrast, anesthetic doses reduce glutamate levels. Early rodent pharmaMRI studies found ketamine‐induced BOLD increases across cortical, cingulate, and hippocampal regions [100]. These effects were attenuated by pretreatment with an mGluR2/3 agonist, suggesting modulation of glutamate release via presynaptic mGluR2 receptors [101]. Human studies showed clear increases in frontal cortical and thalamic BOLD signal [16, 26], which were also attenuated by mGlu2/3 agonists [27] providing a clear translation of the work in animals to humans. While clinical trials with mGlu2/3 agonists have ultimately failed, secondary data analysis suggests a subgroup of patients earlier in the course of their illness had a larger clinical response [102]. This group is known to have glutamate dysfunction, but there were no selection criteria in this domain.

### 7.1 PharmaMRI in Drug Development

Most clinical trials in psychiatry still rely heavily on clinician reports, subjective symptom scales, and self‐report questionnaires to evaluate treatment efficacy. While these tools are indispensable for diagnosing disorders and tracking clinical change, they offer limited insight into the neurobiological mechanisms underlying treatment effects or the brain circuits modulated by a specific compound. Over the past decade, interest has grown in using fMRI to explore such mechanisms, yet pharmaMRI remains underutilized, particularly as a primary outcome in clinical trials. While a review identified > 1300 trials including fMRI, only one‐third used it as the sole primary outcome [103]—mostly to assess pre‐post treatment effects rather than acute pharmacological responses (i.e., pharmaMRI). In fact, a focused review in depression research found that only 6% of fMRI trials qualified as pharmaMRI studies [104].

In the context of clinical studies in psychiatry, pharmaMRI can serve several key purposes, including identification of imaging‐based biomarkers by assessing acute drug‐induced changes in neurobiological systems, validating the theory of drug action, translating mechanistic insights from small animal studies, predicting treatment response at baseline, and stratifying patient populations according to their pharmaMRI response prior to intervention. These applications support both different phases of drug development and the growing move toward precision psychiatry.

In early‐phase trials or experimental medicine studies, pharmaMRI can help establish proof of mechanism (POM) by detecting central nervous system effects in brain regions known to express the molecular target or be disrupted in the disorder of interest. While techniques like PET or SPECT are needed to assess target engagement directly, pharmaMRI provides complementary information by revealing its functional consequences (“functional” target engagement), such as changes in task‐related activity or across large‐scale brain networks regulated by the target receptors. At this stage, dose–response and exposure–response studies are particularly informative, helping to determine whether different drug concentrations lead to linear or nonlinear changes in brain activation. As an example, Javitt et al. [105] conducted a multicentric study directly comparing three candidate neuroimaging biomarkers of glutamate target engagement: resting‐state pharmaMRI, ^1^H‐MRS, and task‐based fMRI. Reversal or blockade of these biomarkers would provide functional evidence of a brain effect for novel glutamatergic agents in schizophrenia. PharmaMRI emerged as the most robust and reliable, with a large effect size (Cohen's *d* = 5.4) and strong cross‐site reproducibility. Importantly, it reflected increased cortical glutamate release following NMDAR antagonism and was sensitive to reversal by mGluR2/3 agonists and other glutamatergic modulators, making it a particularly compelling tool for early‐phase evaluation of glutamate‐based treatments. Importantly, the study also demonstrated its feasibility across major 3 T MRI platforms, provided that proper harmonization and quality control measures are in place. The effects of multiple doses of mGlu2/3 agonists on distributed ketamine‐induced brain changes were assessed with pharmaMRI, aiming to translate the impact of glutamate reduction [27]. Only the highest tolerated doses were sufficient to modulate brain activity.

The optimal dose of drug may not correspond to the highest tolerated dose, due to inverted‐U effects or shifts in the spatial profile of activation related to differential receptor affinity. An example of the inverted‐U effect is the novel negative allosteric modulator of the nicotinic alpha‐7 receptor, which modulates brain activity during fearful face processing at the lower, but not at the higher dose of the drug [106].

In later stages of drug development (Phase II/III), pharmaMRI can help determine whether a compound modulates brain circuits in ways consistent with clinical efficacy, for example, by normalizing disease‐related patterns of brain activity. The NIMH “Fast‐Fail” approach explicitly incorporates POM testing into stage IIA trials to confirm whether target engagement yields measurable neurobiological effects. Conversely, if a compound fails in Phase III despite confirmed target engagement, this approach increases confidence that its effects on brain function are insufficient to produce symptom relief. While not a classic pharmaMRI study, Krystal et al. [107] showed that KOR antagonism increased ventral striatal activation measured with fMRI during a reward anticipation task, compared to placebo, providing a functional biomarker of target engagement in anhedonia. However, closer inspection of their findings suggests that this result was driven by an unusual drop in activity in the placebo group during a moderately reliable task [108], with no change observed following the KOR antagonist. This highlights the importance of careful application of pharmaMRI methods to avoid erroneous conclusions.

Similarly, Konova et al. [109] used a placebo‐controlled, crossover pharmaMRI design to investigate the effects of an acute methylphenidate challenge on resting‐state connectivity in individuals with cocaine use disorder. A single dose of methylphenidate modulated connectivity within the mesocorticolimbic dopamine system, reducing abnormally elevated ventral‐to‐dorsal striatal coupling while enhancing corticolimbic connections, and these changes were associated with addiction severity. While not part of a formal treatment trial, the study illustrates how pharmaMRI can detect short‐term neurophysiological normalization in a clinical population and suggests a potential mechanism by which dopaminergic agents might support behavioral control in stimulant addiction. This kind of experimental medicine approach can help identify circuit‐level markers of responsiveness and inform the repurposing of existing compounds.

### 7.2 Precision Psychiatry: Treatment Response and Confounding Factors

Beyond drug development, pharmaMRI has also been used prior to treatment start to assess to what extent the acute modulation of drug targets can predict clinical response after a treatment series. Two ADHD studies suggest that brain functional changes after a single dose of methylphenidate may predict treatment response. One small study (*N* = 7) linked reduced regional homogeneity in parietal regions to symptom improvement [110], while a larger study (*N* = 56) found that enhanced connectivity between the cerebellar vermis and left precentral gyrus predicted chances of 2‐month clinical response [111]. In MDD, SSRI responders showed reduced activity to fearful (vs. happy) faces in the amygdala, insula, and anterior cingulate after 1 week of treatment. Importantly, these early neural changes predicted later symptom improvement independent of initial mood shifts, suggesting that early correction of negative bias may underlie antidepressant efficacy [112].

Additionally, pharmaMRI has been used to investigate neurobiological changes following prolonged treatment. For example, in ADHD, it served as the primary outcome measure in a clinical trial assessing whether stimulant treatment during development affects the maturation of brain function. Using an oral methylphenidate challenge, the study showed that 4 months of treatment altered the acute pharmaMRI response to methylphenidate in children, but not in adults or a placebo group [113]. As such, pharmaMRI can also serve as a tool to monitor changes in brain responses to compounds during or after treatment, for example, to assess whether neurobiological tolerance to certain compounds develops over time.

It is important to acknowledge that the impact of pharmaMRI results in clinical trials can be influenced by various confounding factors. Comorbid psychiatric or neurological conditions can independently alter brain function and obscure drug effects. Polypharmacy introduces additional pharmacological interactions that may modify or mask the observed responses. Patient heterogeneity, including differences in age, sex, genetics, disease severity, and treatment history, can further complicate the interpretation of pharmaMRI findings and reduce statistical power.

In trial design, pharmaMRI may be most valuable as a proof‐of‐concept biomarker to guide dose selection, identify likely responders, and provide early mechanistic efficacy signals, thereby complementing clinical endpoints and supporting regulatory decision‐making in early‐phase studies.

## 8 Open Science

To accelerate progress in pharmaMRI, the field must embrace open science practices. Large‐scale initiatives in psychiatry, like IMAGEN [114] and ENIGMA [115], have shown the value of data sharing and harmonization, though extending these efforts to functional data, especially in pharmaMRI, remains a challenge. Most pharmaMRI datasets have been collected in specialized centers using in‐house protocols with limited standardization. This has led to small, heterogeneous datasets, limiting statistical power and reproducibility.

A key next step is the sharing of existing data, either through publicly accessible repositories or federated learning approaches that preserve data privacy while enabling large‐scale analyses. Equally important is the development of community‐wide consensus on best practices. Standardization of drug administration paradigms, MRI acquisition protocols, and analysis pipelines would greatly enhance comparability across studies and sites. While standardization across centers and scanner platforms can be challenging, harmonization strategies such as site‐specific calibration, phantom scans, and coordinated preprocessing pipelines can help ensure reproducibility and comparability; separate from post‐processing harmonization [116] which may be challenged by multiple smaller datasets, although we note fMRI datasets typically have internal controls via comparison conditions of tasks and no‐drug or placebo scans in pharmaMRI studies. Data should be curated and shared in standardized formats such as BIDS [117], which already supports BOLD and ASL data structures relevant to pharmaMRI, as well as multimodal data like PET and EEG. Analysis pipelines, currently siloed within individual labs, should be openly documented and made available via platforms like GitHub or the Open Science Framework. Pre‐registration is another underused but essential tool. While a few pharmaMRI studies are pre‐registered, a field‐specific template could improve uptake. Such a template, shared via research societies and conferences, would support transparency and reduce analytical flexibility.

Finally, as pharmaMRI spans both preclinical and clinical domains, open science protocols should be designed with translational relevance in mind, encouraging harmonization not only across human studies but also between species. Implementing these open science practices will enable more reproducible results, larger combined sample sizes, and better cross‐study comparisons, key steps toward clinically useful pharmaMRI biomarkers.

## 9 Future Directions and Conclusion

PharmaMRI is uniquely positioned to advance psychiatry through transdiagnostic, systems‐level approaches aligned with frameworks such as the Research Domain Criteria (RDoC) [118]. Task‐based fMRI, in particular, is inherently suited for assessing multiple functional domains within a single session, supporting the shift from categorical diagnoses toward dimensional, brain‐based phenotyping. Beyond these developments, other emerging pharmacological interventions, including psychedelic‐assisted therapies [119] and orexin receptor antagonists [120], are beginning to be explored with pharmaMRI and represent promising avenues for future research.

Strengthening the translation between preclinical and human work remains a major opportunity for the field. Accumulating evidence shows that homologous large‐scale networks, including resting‐state networks, exist across species [121]. These conserved features of brain organization provide a foundation for cross‐species comparison of drug effects in psychiatry. Indeed, comparable developmental patterns of dysconnectivity in mice and humans are affected by 22q11.2 deletion, a risk gene for different psychiatric disorders [122]. Similarly, another study found strikingly similar patterns of hypo‐ and hyperconnectivity in 20 autism‐linked genetic variants across mice and humans, while also identifying convergent signaling pathways that may underlie these shared patterns [123]. Such studies provide a roadmap for combining cross‐species fMRI with pharmaMRI to test drug effects in mechanistically informed models and to refine the translatability of candidate biomarkers.

Looking ahead, the integration of pharmaMRI with a widening array of complementary modalities is likely to expand, building on current applications as discussed in Section 5. In the coming years, additional techniques such as functional MRS (fMRS) and functional ultrasound (fUS) are poised to play an increasingly important role in refining mechanistic specificity. fMRS provides dynamic neurochemical measurements, for instance, changes in glutamate and lactate, that can be acquired alongside hemodynamic responses. A combined fMRS/fMRI study demonstrated that ketamine administration induced time‐dependent increases in Glu and Lac in the anterior cingulate cortex, which occurred alongside but were temporally dissociated from BOLD signal changes [23]. Moreover, Jelen et al. [49] showed that pre‐treatment with naltrexone reduced ketamine‐induced increases in glutamate + glutamine in patients with major depressive disorder, suggesting a functional interaction between opioid and glutamatergic systems. In animal models, fUS has emerged as a high‐spatiotemporal‐resolution technique capable of imaging CBV changes in freely moving [124] or minimally restrained animals [125]. This technology could complement both anesthetized and awake fMRI in preclinical pharmaMRI studies, enabling more naturalistic assessments of drug effects.

A critical priority is improving the reliability of drug‐induced MRI measures. While quantitative CBF measures such as ASL show high test–retest reliability (e.g., intraclass correlation coefficients > 0.8 in gray matter), BOLD fMRI shows more variability. Some studies have reported good reliability for specific tasks, such as the n‐back [108, 126, 127], but subsequent work has cast doubt on their suitability for individual‐differences research [128]. Even less studied is the reliability of drug effects on the MRI signal. This is of particular relevance for drug models of disease or mechanism, where changes in signal in response to a pharmacological challenge are used to validate target engagement or brain‐based mechanisms. When examined, drug‐related signal changes have shown moderate reliability overall, but this can vary across brain regions [16, 129].

Finally, integrating pharmaMRI with large‐scale functional imaging datasets could help overcome current sample‐size limitations and improve interpretability at the individual level. For instance, cumulative resting‐state connectivity patterns from large cohorts have been linked to clinical symptoms [130] and could serve as baselines for assessing medication effects. Reuse of population imaging data also offers the opportunity for pharmaMRI. For example, a recent study mapped drug‐induced functional reorganization onto in vivo neurotransmitter gradients, revealing many‐to‐many relationships between drug effects and regional neurotransmitter distributions [131]. Building on preclinical work using “domain gauges” to profile multivariate drug signatures, these approaches lay the foundation for a translational framework connecting molecular targets to systems‐level dynamics [132]. In parallel, integrating normative modeling into receptor‐informed pharmaMRI analysis would allow researchers to move beyond group averages to quantify individual deviations from typical connectivity [133], enabling the development of biomarkers that reflect both molecular engagement and individual‐level systems dysfunction, key for advancing mechanistically informed, personalized treatments.

## Conflicts of Interest

Christin Y. Sander and Anouk Schrantee declare no conflicts of interest. Tudor M. Ionescu is an employee of Boehringer Ingelheim Pharma GmbH & Co KG. Ottavia Dipasquale is an employee of Olea Medical. Mitul A. Mehta has research funding from Nxera and Lundbeck and received in‐kind contributions from Compass Pathways. He has consulted for Boehringer Ingelheim and Nxera and received speaker fees from Takeda.
